# Pediatric Langerhans Cell Histiocytosis: An Aggressive Presentation

**DOI:** 10.7759/cureus.25684

**Published:** 2022-06-06

**Authors:** Shasikala Suthersan, Fei Ming Ong, Thevagi Maruthamuthu, Chenthilnathan Periasamy, Bee-See Goh

**Affiliations:** 1 Otorhinolaryngology - Head and Neck Surgery, Universiti Kebangsaan Malaysia Medical Centre, Kuala Lumpur, MYS; 2 Otorhinolaryngology - Head and Neck Surgery, Penang General Hospital, Penang, MYS

**Keywords:** imaging, pediatric, maxillary sinus, temporal bone, langerhans cell histiocytosis (lch)

## Abstract

Encountering a young child with an enlarging painless facial swelling often raises concerns in the treating physician about the possibility of a congenital lesion or an unfavorable pediatric tumor. We discuss a case of a female child who presented with multiple craniofacial swellings, which turned out to be Langerhans cell histiocytosis (LCH). She was subsequently diagnosed with multisystem LCH (MS-LCH) with risk-organ involvement, which included the craniofacial bones, skin, hemopoietic system, and liver. We analyze the various presentations and systemic complications of this rare pediatric tumor, LCH, with an aim to address the diagnostic dilemma associated with this great masquerader.

## Introduction

In the pediatric population, a rapidly progressing facial swelling may imply sinister diseases such as metastatic neuroblastoma, Ewing sarcoma, osteogenic sarcoma, rhabdomyosarcoma, as well as Langerhans cell histiocytosis (LCH) on the list of differentials [1]. Although LCH is a rare condition, with an overall incidence estimate of 4.6 cases per million children under 15 years of age, it is often associated with long-term sequela and mortality [2]. LCH is a clonal proliferative disease and is characterized by the pathological accumulation of Langerhan cells in the bones, skin, liver, spleen, lungs, bone marrow, and brain [2-3]. There is a large spectrum of clinical presentations in pediatric LCH, ranging from an isolated lesion to multisystem disease [3-4]. In this report, we highlight the importance of early recognition of different types of clinical manifestations of LCH in order to expedite the diagnosis and commence targeted treatment.

## Case presentation

**Figure 1:**
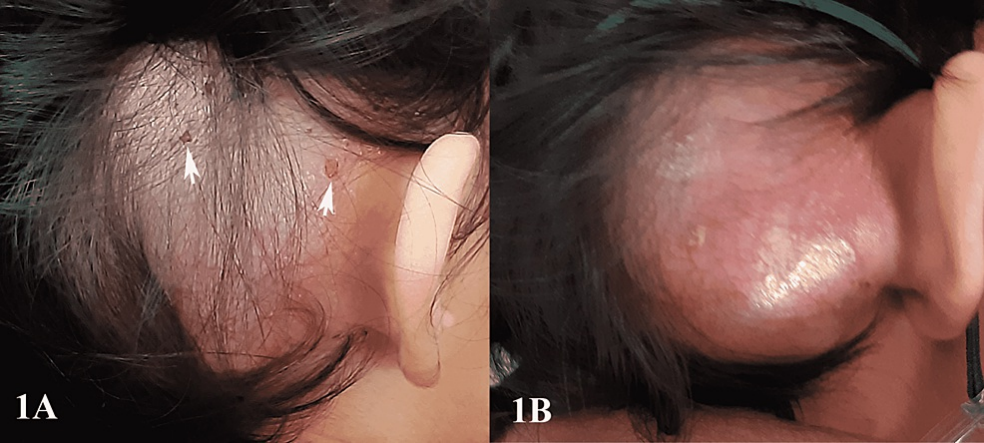
Photos illustrating patient's condition - 1 Figure 1A demonstrates the right postauricular swelling with the presence of flaky cutaneous lesions (white arrow) on the scalp. Figure 1B shows worsening erythema and rapid enlargement of swelling after four days

The patient was a one-year-old Malay girl who presented with a right postauricular swelling, which had progressively increased in size over a period of three months. It was a painless, fluctuant, cystic-like swelling and measured 7 x 7 cm (Figure 1).

**Figure 2:**
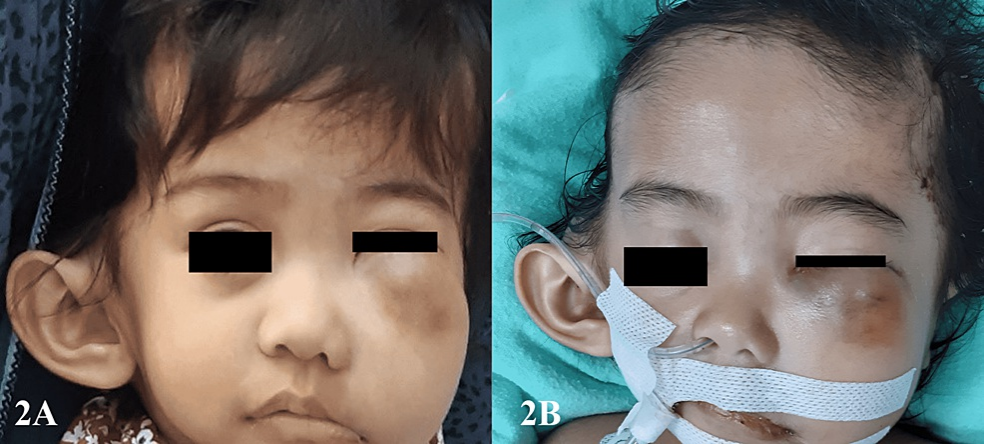
Photos illustrating patient's condition - 2 Figure 2A shows the left maxillary swelling with skin discoloration and the right pinna pushed outward and laterally. Figure 2B shows the postoperative mild reduction in left maxillary swelling following tumor debulking via the sublabial approach

There was no associated ear pain, ear discharge, or reduced hearing. The patient had also developed left lower eyelid ecchymosis, which had progressed into a large maxillary swelling over a period of two weeks. The left maxillary swelling measured 4 x 4 cm and was firm in consistency (Figure 2). She also had multiple flaky lesions over the scalp, which were itchy. There was a clinically palpable liver but no evidence of splenomegaly or generalized lymphadenopathy was palpable. The child exhibited signs of failure to thrive with a current weight of only 8 kg, despite a birth weight of 2.7 kg. The girl’s condition rapidly deteriorated over a period of one week, with a spiking fever, tachycardia, and increased lethargy and irritability. She also had normochromic normocytic anemia, hypoalbuminemia, and ultrasound-proven hepatomegaly.

CT of the brain, orbit, and paranasal sinuses and the high-resolution image of the temporal bone showed a heterogeneous lesion on the right postauricular region measuring 6.9 x 5.1 x 7 cm, with bony erosion of the right temporal and right mastoid air cells. There was an intracranial extension of the mass into the right temporal region and right posterior fossa. The mass closely abutted the right tentorium cerebelli and the sigmoid sinus; however, the sinus remained patent. There was an extension of the mass into the external auditory canal and the middle ear but with intact ossicles. Another similar heterogeneous mass, measuring 4.6 x 3.4 x 4.1 cm, was noted at the left infraorbital region with infraorbital extension and associated bony erosion involving the orbital floor. There was evidence of bony erosion involving all walls of the left maxillary sinus and the left alveolar process. The mass completely obliterated the left maxillary sinus. The presence of a clear fat plane with the left orbit and left extraocular muscles and left optic nerve intact was also observed.

The patient was scheduled for an MRI; however, she continued to have clinical deterioration with rapid enlargement of the mass. In view of the urgency to obtain a tissue biopsy, a multidisciplinary discussion involving otorhinolaryngology, neurosurgery, and pediatric oncology deemed the maxillary mass to be a safer option for biopsy and with less intraoperative risk. This was based on the CT imaging showing the postauricular mass with the presence of intracranial extension, and hence this surgical approach was not used in view of the risk of cerebrospinal leak and intracranial complications. The child underwent nasal examination under anesthesia and incisional biopsy of the left maxillary tumor via sublabial approach. Intraoperatively, it was noted that the sublabial mucosa was intact but the sulcus was obliterated. The capsulated tumor broke during sublabial incision and plenty of stale blood content was noted within the tumor. The tumor was dark red in color with a jelly-like consistency. There was tumor extension and erosion of the left alveolar process of the maxilla, and anterior, lateral, and medial maxillary walls.

**Figure 3:**
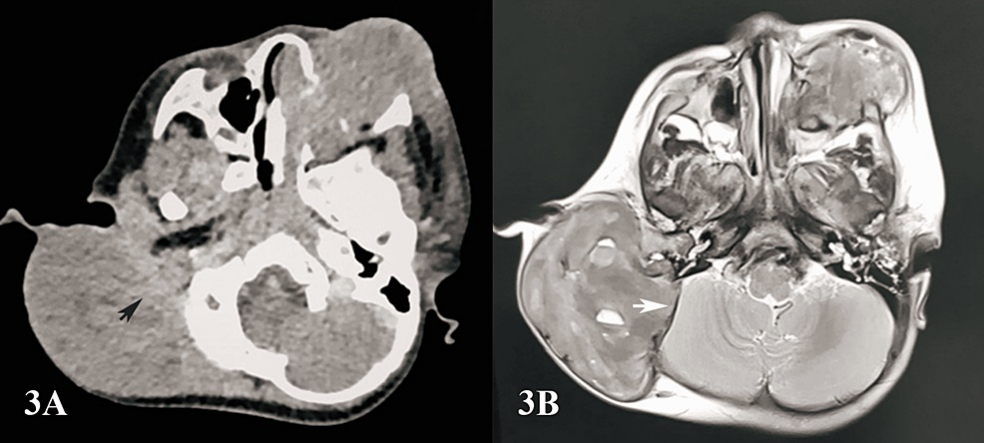
CT and MRI findings Figure 3A: CT of the brain and paranasal sinuses illustrating poor demarcation between the mass at the right temporal region with the intracranial fossa (black arrow). Figure 3B: MRI of the brain showing the clear demarcation (white arrow) between the mass at the right temporal region and posterior cranial fossa CT: computed tomography; MRI: magnetic resonance imaging

The patient then underwent an MRI of the brain, which demonstrated the skull base mass at the right temporal region arising from the bone. The lesion was heterogeneous hyperintense on T1W1 and iso-hypointense on T2W1. There was a presence of cystic components within, with the fluid level seen. The mass measured 5.7 x 7.0 x 7.5 cm. The mass was hypointense on gradient recalled echo (GRE) and there was a heterogeneous enhancement on post-gadolinium images. There was no intracranial extension. The sigmoid sinus was compressed by the mass; however, it remained patent. A similar appearance was noted at the left maxillary lesion measuring 2.7 x 3.5 cm, with mass effect onto the left orbital floor and stretching the left inferior rectus muscle. Differentials listed included metastasis (especially neuroblastoma), a less likely differential in lymphoma or rhabdomyosarcoma (Figure 3).

The histopathological findings revealed LCH. Microscopy showed tumor tissue formed by diffuse patternless sheets of large atypical mononuclear cells with moderate to abundant amounts of eosinophilic to vacuolated cytoplasm with binucleation and multinucleation seen. Admixed with these atypical cells were a variable number of eosinophils, neutrophils, lymphocytes, and plasma cells. Immunohistochemistry revealed many atypical mononuclear cells expressing CD1a, CD68, and S100. The cells stained negative for ALK (anaplastic lymphoma kinase for neuroblastoma, non-small cell lung cancer, and anaplastic large cell lymphoma), CD3 (ruling out T-lining lymphoma), CD20 (ruling out B-lineage lymphoma), CD30 (for lymphoid malignancies or germ cell tumors), EMA (epithelial membrane antigen for soft tissue tumors), and myogenin (ruling out rhabdomyosarcoma).

The patient was started on six cycles of vinblastine and oral prednisolone. She, unfortunately, had a poor response after initial chemotherapy and has been reclassified as LCH with disease progression and multisystem involvement and is currently undergoing second-line treatment with a combination of oral prednisolone, cytarabine, and vincristine. She has shown a favorable response to the latest regime and continues to receive multidisciplinary care from the pediatric, oncology, otorhinolaryngology, and dermatology teams.

## Discussion

LCH is a heterogeneous disease with a wide spectrum of clinical presentations, primarily depending on the organ or systems involved [3,5]. The clinical presentation can be broadly divided, based on the extent of involvement, into single-system LCH (SS-LCH) and multisystem LCH (MS-LCH), and the presence of risk organs [5]. Risk organs, which include the hematologic system, bone marrow, spleen, and liver, are associated with higher mortality and poorer prognosis [5]. It is estimated that two-thirds of children with LCH have single system manifestation with the remaining one-third, usually of a younger age, having multisystem presentation [3]. Based on the study by Haupt et al. (2013), the three most common organs involved are bone (80%), skin (33%), and pituitary (25%). The liver, spleen, hematopoietic system, and lungs are involved in 15% of cases; the involvement of lymph nodes (5-10%) and the central nervous system excluding the pituitary (2-4%) has also been reported [5].

Bone is the most common organ involved in LCH, with the skull vault most frequently involved in younger children, who usually present with a painless fluctuant lump [5-7]. Our patient had one lesion affecting the temporal bone, and this manifested as a fluctuant postauricular swelling with a palpable rim over the bony defect whereas the maxillary sinus bony lesion was firm in consistency. Although both the swellings were part of the same disease, they exhibited different consistency due to the bony location. Temporal bone LCH can present with postauricular swellings, refractory recurrent otorrhea, otalgia, aural polyps, or hearing impairment [8-9]. We highlight that in the pediatric group, there must be a lower threshold for imaging as compared to adults, and procedures such as aspiration of a cystic-like swelling or biopsy of an aural polyp should only be undertaken after appropriate imaging due to the likelihood of encountering rare pediatric tumors or congenital lesions. The maxillary sinus lesion in our patient showed periorbital ecchymosis, which could have been easily mistaken for a non-accidental injury [10]. Other examples of bony lesions are orbital lesions with proptosis, mandibular lesions with “floating” teeth, and long bone or vertebral lesions with musculoskeletal pain [7,10].

Cutaneous manifestations of LCH are diverse and represent the second most common presentation of LCH. They can manifest as scalp seborrheic dermatitis or a non-specific rash (vesicular, ulcerative, eczematous, or papular) over the scalp and trunk [6,7]. It is important to note that these cutaneous lesions are indicative of an underlying multisystem disease, with only 2% of cutaneous LCH being diagnosed as SS-LCH [5,7]. We emphasize the need for a clinician to tie up and correlate skin conditions and facial swellings to be part of a larger spectrum of disease rather than considering them as two separate entities. Localized cutaneous LCH is known to have self-resolving tendencies, with the congenital variant of self-regressing cutaneous LCH being termed as Hashimoto-Pritzker type [11].

Risk-organ involvement can be revealed by blood investigations showing cytopenia, liver dysfunction, and imaging-proven hepatosplenomegaly [7]. Anemia and thrombocytopenia, as demonstrated in our patient, are among the criteria required to confirm hematopoietic involvement. Hemoglobin levels between 10 and 7 g/dl are considered mild whereas those below 7 g/dl are considered severe, on the condition that other causes, such as iron deficiency, have been excluded. Mild thrombocytopenia is indicated by platelets between 100,000 and 20,000/mm^3^ whereas severe thrombocytopenia means platelets below 20,000/mm^3^. Hepatomegaly or splenomegaly requires the ultrasound confirmation of the liver or spleen being palpable 3 cm below the costal margin at the mid-clavicular line. Liver involvement can also be shown by deranged parameters such as hyperbilirubinemia, hypoalbuminemia, and raised ALT (SGPT) and AST (SGOT) [5].

CT scan is required to confirm the presence of the bony lesion and delineate the extent of osseous and cortical destruction as well as soft-tissue involvement [12]. The role of MRI is important in demonstrating intracranial extension, bone marrow involvement, and associated soft-tissue mass or inflammation in LCH of the bone [12]. This was evident in our patient where the MRI clearly demonstrated the absence of intracranial extension of the mass as compared to the CT findings which stated otherwise. The typical LCH findings on imaging include skull lesions with punched-out beveled edges, geographic skull, and the “floating tooth” sign secondary to alveolar destruction [13]. Other findings include vertebra plana, atypical lung cysts, and an absent posterior pituitary bright spot with infundibular thickening [13]. The patient underwent imaging, which demonstrated a similar punched-out beveled lesion on the temporal bone and left alveolar erosion corresponding to the maxillary lesion.

As LCH is essentially a histological diagnosis, one important step in the management involves obtaining a tissue biopsy [5]. Our patient had two craniofacial swellings, and a careful multidisciplinary discussion deemed the maxillary swelling via a sublabial approach to be the safer option to obtain tissue biopsy as compared to the temporal lesion, which had a higher risk of complications and morbidity due to its proximity to the brain. The histologic characteristic of LCH is a proliferation of the Langerhans’ dendritic cell in a background of inflammatory cells with sheets of eosinophils, as well as the presence of Birbeck granule on electron microscopy [14]. The current standard of diagnosis is positive immunohistochemical staining of S100 and CD1a or CD207 [5,14].

The mainstay of treatment of LCH is the use of chemotherapy and prednisolone. MS-LCH is treated with standard therapy involving an initial six-week course of prednisolone (40 mg/m^2^/day orally for four weeks followed by tapering over two weeks) and vinblastine (6 mg/m^2^ weekly intravenous bolus). The maintenance phase of therapy depends on the response to initial therapy, which is an important prognostic marker. Non-intensive maintenance therapy, with a one-year regime of prednisolone, vinblastine, and oral mercaptopurine, is advocated for patients with complete disease resolution or minimal disease in non-risk organs. On the contrary, a second initial course (similar to the first one but with corticosteroids given for three days a week) is recommended for patients with a risk disease with evident but incomplete response in risk organs, as well as for patients with a low-risk disease without improvement after the first course. Risk-group patients with a lack of improvement in risk organs after 6-12 weeks of therapy are candidates for salvage options. The adverse prognostic markers include multisystem presentation, risk-organ involvement, and poor response to initial therapy [15].

## Conclusions

LCH is a great masquerader, with presentations ranging from a simple isolated cutaneous lesion to a mistakenly diagnosed nonaccidental injury with periorbital ecchymosis to a possibly fatal multi-organ failure secondary to disseminated disease. When dealing with children with rapid progressive craniofacial swellings, a high index of suspicion is needed even for rare conditions such as LCH. There must be an emphasis on a careful head-to-toe evaluation of patients in order to tie up and correlate multisystem presentations, and appropriate early imaging is of paramount importance to prevent delayed diagnosis and treatment. This provides a better prognosis for these children and a glimmer of hope for the parents.
